# Predicting the power of informal learning opportunities on technology use of physical education teachers

**DOI:** 10.3389/fspor.2025.1653809

**Published:** 2025-10-20

**Authors:** Jonas Wibowo, Rüdiger Hofmann, Tabea Brand, Hendrik Wiese

**Affiliations:** ^1^ Faculty of Sport Science, Ruhr-Universität Bochum, Bochum, Germany; ^2^ Faculty of Human and Social Sciences, Bergische Universität Wuppertal, Wuppertal, Germany; ^3^ Faculty of Educational Sciences, Universität Hamburg, Hamburg, Germany

**Keywords:** digital technology, technology acceptance, UTAUT, informal learning, professional development (PD), workplace learning, structure equation modeling (SEM)

## Abstract

**Introduction:**

This study investigates the factors influencing the acceptance of digital technologies among physical education (PE) teachers, aiming to support the integration of contemporary digital tools into movement-based learning. Drawing on the Unified Theory of Acceptance and Use of Technology (UTAUT), the research explores how contextual and personal factors shape teachers' willingness and ability to use digital technologies in their professional practice.

**Methods:**

A quantitative survey was conducted with 239 physical education teachers. The collected data were analysed using structural equation modelling (SEM) to identify relationships among the UTAUT constructs and additional variables, including formal and informal learning opportunities, age, and gender.

**Results:**

The analysis revealed that supportive conditions and behavioural intention equally predicted the actual use of digital technologies. Perceived usefulness and social influence significantly affected behavioural intention, whereas perceived ease of use showed no significant effect—suggesting a generally high baseline of digital literacy among the participating PE teachers. Informal learning opportunities exerted a stronger influence on performance expectancy and effort expectancy than formal learning opportunities. Age and gender were not significant predictors of technology acceptance.

**Discussion and Conclusion:**

The findings indicate that digitalisation is increasingly embedded in educational practice across demographic groups. To enhance digital technology integration in PE, stakeholders should invest in both digital infrastructure and teacher development, prioritising informal, collaborative, and practice-oriented learning environments that foster sustained technology use in movement-based education.

## 1 Introduction

The acceptance of digital technologies by physical education (PE) teachers may be seen as one element of contemporary PE. For example, we find numerous examples of how digital technologies can be implemented to foster students' learning or help teachers organise themselves (1, 2). In saying this, we do not imply that every single use of digital technology guarantees high-quality teaching and learning or that every use is to be regarded as pedagogically meaningful (3). From our perspective and based on the literature, we wish to provide certain reasons why digital technologies may be meaningful in PE. Digitalisation and the growth of a culture of digitality (4) are having such a huge impact that the culture in our society is changing, even the movement culture (5, 6). Examples of the many small changes are the already enormous and still-growing number of video tutorials on learning a movement, tracking technologies and training processes as well as the worldwide connection of people with special interests via social media.

Academics in sport pedagogy argue that physical education should prepare students to participate in the culture of movement, promote individual self-cultivation or movement education, encourage lifelong physical activity, and cultivate general competencies in line with societal values (7–9). In German sport pedagogical debates, this is referred to as education in and through sport, play and movement (9, 10). Digital technology can be considered legitimate if it reflects established cultural (digital) practices within movement culture and facilitates self-cultivation. Participation in this movement culture involves making responsible, reflective choices rather than simply replicating existing practices.

Digital technology also has legitimacy when it effectively supports students' learning processes, as the development of knowledge and skills provides the basis for responsible decision-making. According to learning theory, increasing engagement and optimising time spent on meaningful activities can significantly improve learning outcomes (11). In addition, the quality of these activities is crucial (12, 13). Other theories emphasise media support for the acquisition of motor skills (14) or social environments that foster autonomy (15). There are more reasons to legitimise the use of digital technologies in PE, including the development of media competences (16). However, the two reasons mentioned above—educational goals and learning outcomes—are central from our perspective. Given that digital technologies are not only legitimate, depending on how they are used and with what justification, but also a central aspect of contemporary PE, PE teachers' acceptance of such technologies is fundamental. We are not advocating for a maximisation of technology acceptance but rather for a critical acceptance and openness towards technologies in educationally legitimised teaching scenarios.

This paper elaborates on what influences PE teachers' behavioural intention (BI) and use behaviour (UB) as two aspects of technology acceptance with regard to different digital technologies. We use the unified theory of acceptance and use of technology (UTAUT) to identify and model influencing factors. Furthermore, we aim to identify interconnections with the development aspects of other professional competences.

### 1.1 Influence of internal and external factors on behavioural intention

The increasing prevalence and accessibility of digital technologies have prompted researchers to examine teachers' receptivity and utilisation of these tools in the classroom (17). In recent decades, many models have been proposed to describe the mechanisms and factors influencing technology adoption. Notable examples are the UTAUT and the technology acceptance model (TAM). The UTAUT model is a prominent model for understanding the factors contributing to individuals' acceptance and use of technology (2). The UTAUT (18) postulates that there are four fundamental determinants of user intention and actual use of technology: performance expectancy (PE), effort expectancy (EE), social influence (SI) and facilitating conditions (FC). It is hypothesised that the effects of these determinants are moderated by individuals' gender, age and experience and the voluntariness of technology use (19). The UTAUT model has been extensively employed in consumer technology, substantiating its efficacy in forecasting and elucidating technology acceptance (20, 21).

However, the UTAUT model's application in the context of educational research, particularly among PE teachers, remains scarce. There is a paucity of empirical data on teachers' technology acceptance (22). The extant literature indicates that the adoption and implementation of digital technologies in PE remains a complex and multifaceted issue (23). While some digital technologies are more widely used by physical educators, various external and internal barriers can impede their integration. External barriers encompass constraints such as limited time, insufficient expertise and inadequate resources. For example, teachers have noted that the brief duration of PE classes leaves little opportunity for the effective incorporation of digital tools, while a lack of dedicated training and limited access to up-to-date technological resources further restrict the implementation process. By contrast, internal barriers are primarily related to teacher beliefs and the adherence to established pedagogical practices (24). A further avenue for research would be to adapt the UTAUT model to investigate the acceptance of technology by PE teachers. This could provide valuable insights into the factors influencing their adoption and use of educational technology (17). Educators' perceptions of the feasibility, technical competence and availability of support can significantly influence their willingness to incorporate digital technologies into their teaching practices (25).

### 1.2 Differences between teachers in their use of digital technologies

According to the German sample of the International Computer and Information Literacy Study (ICILS) 2023, more than 70% of participating teachers used digital technologies in the classroom at least once a day. The international average of 61% is significantly lower (26). Even in ICILS 2018, 55% of participating German teachers stated that they had been using digital technologies in the classroom for over five years (27). The type of use is very diverse, but computer-based information sources are used most frequently (27). These data refer to the German sample and all teachers, not just PE teachers. Therefore, the next step is to learn more about PE teachers' use of digital technology.

To demonstrate the variety of options available to PE teachers, various authors have attempted to summarise them. Wibowo et al. (6) compiled list of product categories, which included the following seven categories: presentation tools, learning management systems, data exchange tools, production tools, movement-related tools, databases and research tools. Similarly, Jastrow et al. (2) described different uses to better relate different studies on the use of digital technologies in PE and their benefits and limitations, listing six options for use: recording data, video evaluation, tagging movement, game situations, creating media products, and wikis (2). Some of these seem to correspond to the categories of Wibowo et al. (6), such as production tools and the creation of media products. However, there are major differences between the two versions. For example, Jastrow et al. (2) did not list any learning management systems or data exchange tools. Well-planned use in PE lessons, regardless of the use type, can benefit pupils and teachers (1). Teachers can make their lessons more student-centred, with developmentally appropriate feedback and more effective learning experiences (1). For students, digitally based PE lessons can increase their digital literacy, video evaluation can optimise sport-specific motor skills and abilities and increase game- and sport-related knowledge, and game situations can make PE lessons more interactive and motivating while also appealing to challenging students (1, 2). When analysing the use of digital technologies, a distinction should be made between different types, as there are apparent differences in their benefits.

Besides the findings on the types and frequency of usage of digital technologies, some studies have indicated that teachers differ significantly in their use of these technologies in accordance with specific properties. In the German teacher sample of the ICILS 2018, there was a significant difference between teachers below the age of 49 (the younger teachers) and the group of older teachers (>50), as teachers under 50 used digital technologies more frequently (28). Further findings from the same study showed no differences concerning teachers' daily use in relation to gender or experience of using digital technologies.

### 1.3 Influence of different learning opportunities

Integrating digital technologies also depends on teachers' professional development (29, 30). Formal learning opportunities (29, 31), as well as informal learning activities (32–34), are considered important factors. Experts from the field of adult learning assume that learning in work–life occurs predominantly informally (35, 36). It has been repeatedly stated that formal learning opportunities often involve transferring abstractly acquired knowledge to concrete applications in everyday work–life situations (37–39). Within informal learning opportunities, this problem should occur less, as this type of learning is structured through concrete everyday work–life problems and finding solutions for them (34, 40, 41). Concerning the acceptance of digital technologies by PE teachers, informal learning opportunities should have a stronger impact on the person's internal factors (PE, EE) than formal learning opportunities.

According to the mainly non-PE findings, it can be assumed that the UB of PE teachers differs in terms of the frequency of use and how these technologies are used. The influencing factors can be discerned as both internal (PE and EE) and external (SI and FC) factors, while the internal factors are probably influenced by the formal and informal learning opportunities (FLO and ILO).

## 2 Research questions and hypotheses

**Figure 1:**
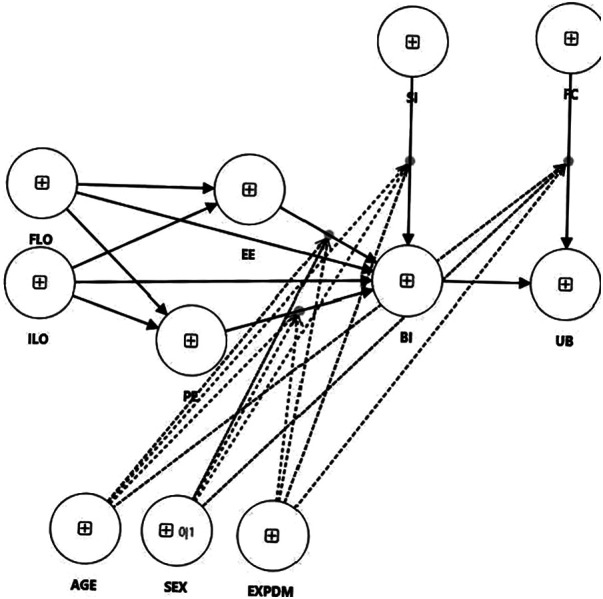
Research model (EXPDM = experience with digital media).

Based on these findings, we formulate three main research questions (RQs) and 19 hypotheses (Figure 1).

• RQ1: What influences the use behaviour (UB) of physical education teachers with regard to digital technologies?
  ○ H1.1 Age has a negative effect on UB.
  ○ H1.2 There are no differences between genders in UB.
  ○ H1.3 Experience has no effect on UB.
  ○ H1.4 FC have a significant influence on UB.
  ○ H1.5 The effect of FC on UB is stronger for older and more experienced teachers.
  ○ H1.6 BI has a significant influence on UB.
• RQ2: What influences the behavioural intention (BI) of physical education teachers towards digital technologies?
  ○ H2.1.1 PE has a significant influence on BI.
  ○ H2.1.2 The effect of PE on BI is larger for men than for women.
  ○ H2.1.3 The effect of PE on BI is larger for younger than for older teachers.
  ○ H2.2.1 EE has a significant influence on BI.
  ○ H2.2.2 The effect of EE on BI is larger for women than for men.
  ○ H2.2.3 The effect of EE on BI is larger for older than for younger teachers.
  ○ H2.2.4 The effect of EE on BI is larger for less experienced than for more experienced teachers.
  ○ H2.3.1 SI has a significant influence on BI.
  ○ H2.3.2 The effect of SI on BI is larger for women than for men.
  ○ H2.3.3 The effect of SI on BI is larger for older than for younger teachers.
  ○ H2.3.4 The effect of SI on BI is larger for less experienced than for more experienced teachers.
• RQ3: Do different learning opportunities influence the internal factors relating to the technology acceptance of physical education teachers?
  ○ H3.1 ILO have a stronger positive effect on PE than FLO.
  ○ H3.2 ILO have a stronger positive effect on EE than FLO.

## 3 Materials and methods

### 3.1 sampling strategy and participants

To validate the delineated model, the current study conducted an empirical analysis based on an online survey of regular PE teachers, focusing on those teaching Grades 5–13. The recruitment was executed using a two-step strategy. First, emails with an invitation were sent to all school secretaries of all relevant schools (*n* = 1,167) in North Rhine–Westfalia (186 lower secondary schools, 358 comprehensive schools and 623 grammar schools). After a relatively low response of 168 teachers, the second step was to post calls for participation on social media platforms (Facebook groups for PE secondary teachers and the Instagram channel of a major influencer in the PE area). Despite bias concerns, this strategy was chosen for economic reasons (see the Limitations section).

Thus, 239 PE teachers (125 females and 114 males) from various school types (121 grammar schools, 77 comprehensive schools, nine secondary schools, seven lower secondary schools and 25 other schools) were surveyed (see Table 1). The mean age of participants was 41.8 years (*M* = 41.8, *SD* = 11.2, min. = 20, max. = 68), and the mean teaching experience was 13.7 years (*M* = 13.7, *SD* = 9.9, min. = 1, max. = 40). The average experience of using digital technologies for PE was 4.3 years (*M* = 4.3, *SD* = 4.5, min. = 0, max. = 31).

**Table 1:** Demographic variables for the teacher sample.

Demographics		*n*	%
Age	≥50	68	28.5
	<50	171	71.5
Gender	Male	114	47.7
	Female	125	52.3
Experience using digital tools	0–10	216	91.1
	>10	23	8.9
School type	Grammar school	121	50.6
	Comprehensive school	77	32.2
	Secondary school	9	3.8
	Lower secondary school	7	2.9
	Other	25	10.5

### 3.2 Instruments

This study measured eight latent variables, using reflective measurement scales. Items were measured on a 5-point Likert scale.

The variables for the UTAUT model (PE, EE, FC, SI and BI) were adapted from Venkatesh et al. (19), taking into account the German translation in Nistor, Wagner and Heymann (42) and the subject of PE (see Supplementary Table 1).

UB was measured using the nine product categories (presentation tools, learning management systems, cloud services, production tools, movement-related tools, searching tools, social media, messenger, and orientation tools) from Wibowo et al. (6) and the 5-point ordinal scale from ICILS 2018: never; less than once a month; at least once a month, but not every week; at least once a week, but less than daily; daily (27). The integration of the usage of all product categories meant that this was not a mere representation of the quantity of technology use but also a representation of the variety of use.

The construct of ILO was measured using three items derived from Kyndt et al. (33). Due to economic reasons, only these three items were considered, as they were assumed to be most important for PE teachers. The construct of FLO was measured by asking for the number of professional development courses taken within the last two years (see Table 2).

**Table 2:** Descriptive statistics of the standardised latent variables.

Construct	Med.	Min.	Max.	Kurtosis	Skewness
BI	.03	−1.72	1.46	−1.108	−.152
EE	.18	−2.74	1.36	.068	−.769
FC	−.10	−2.15	1.74	−.881	.078
FLO	−.51	−.51	4.79	7.902	2.645
ILO	−.27	−1.66	2.93	−.017	.599
PE	.05	−2.89	1.62	−.119	−.520
SI	−.18	−2.21	1.84	−.849	.050
UB	−.14	−1.96	3.39	.568	.857

The descriptive statistics supported the assumption of normal distributions. The kurtosis observed oscillated between a minimum of −1.108 for the BI scale and a maximum of 7.902 for FLO, while the asymmetry values ranged from −0.769 (EE, min.) to 2.645 (FLO, max.).

### 3.3 Methods

The dataset was evaluated using structural equation modelling (SEM). This method was used because of the following advantages compared to other evaluation methods (43):

• SEM multivariate analyses of causal models can be performed, and the effects of the independent and dependent variables can be estimated simultaneously.
• Models can be constructed that contain latent factors and constructs.
• Free determinants or parameters that cannot be derived by assumptions can be estimated simultaneously in structural equation models.
• Measurement errors can be considered or corrected so that the reliability of the model analysis increases.
• Due to improved estimation algorithms, non-multivariate normally distributed variables can also be considered in the models.

A covariance analysis approach was also chosen within the SEM, as it is particularly suitable for empirical examination of a theoretically based hypothesis system (44). However, a variance analysis approach is preferable if no theoretical or logical models can be derived (44).

We estimated our model by using partial least squares structural equation modelling (PLS-SEM) and SmartPLS 3.0 software (45). According to Chin (46), PLS-SEM estimation provides several benefits compared to covariance-based methods, especially when testing complex structural models (47). Among other benefits, PLS-SEM maximises the explained variances, increases prediction accuracy and does not make strict distributional assumptions. PLS-SEM is primarily used to develop theories in exploratory research (47). Moreover, mediation, moderation and multi-group analysis can be performed using PLS-SEM. This study applied a mean-replacement approach for missing values (<1%) and a conservative *no sign changes* bootstrapping procedure based on 5,000 bootstrap runs. The bootstrapping results can be used to test hypotheses by using p-values, t-statistics or *t* values. It can be inferred that the link examined is significant at the 5% level, using a significance threshold of 0.05 (=5%, *p* < 0.05). T-statistics are also compared to t-table or crucial values for one-tailed testing, with a significance level of 0.05 (=5%) of 1.65. As a result, if the t-statistics or *t* values are greater than 1.65, the hypothesis value is acceptable.

Using SmartPLS 4.1 software, PLS-SEM was used to model and analyse the data. The PLS path model was made up of two parts: (i) the structural model (also known as the inner model in PLS-SEM), which shows the correlation between latent variables/constructs; (ii) the measurement model (also known as the outer model in PLS-SEM), which shows the correlation between latent variables/constructs and their indicators.

### 3.4 Measurement model assessment

All constructs in the research model were first-order reflective.

Cronbach's alpha is a measure of the internal consistency or reliability of a construct, that is, how closely related the set of items comprising the construct are as a group. The result is usually a number from 0 to 1, but a negative Cronbach's alpha can also occur, suggesting that something is seriously wrong with the operation (e.g., if some score items have reversed polarity relative to others, the mean of all the inter-item correlations can be negative: The polarity of the items should always be aligned). The general guidelines on Cronbach's alpha for construct reliability and validity are as follows: Below 0.60 is unacceptable, 0.60–0.70 is minimally acceptable, 0.70–0.80 is respectable, 0.80–0.90 is very good, and above 0.90 is strong (48).

The Fornell–Larcker Criterion was used to determine discriminant validity. Discriminant validity determines whether the constructs in the model are highly correlated among themselves. It compares the square root of the average variance extracted (AVE) of a particular construct with the correlation between that construct and other constructs. It is generally suggested that the square root of the AVE should be higher than the correlation of the construct with others. Otherwise, the individual construct does not provide much discrimination (i.e., unique explanatory power).

Table 3 depicts the reliability and validity statistics and the factor loadings.

**Table 3:** Reliability and validity statistics.

C	Discriminant Validity: Fornell–Larcker Criterion									
	Cronbach's Alpha	(1)	(2)	(3)	(4)	(5)	(6)	(7)	(8)	(9)
(1) BI	0.84	0.87								
(2) EE	0.91	0.42	0.88							
(3) EXPDM	N/A	0.12	0.04	1.000						
(4) FC	0.73	0.41	0.59	0.06	0.81					
(5) FLO	N/A	0.33	0.13	0.15	0.27	1.000				
(6) ILO	0.71	0.57	0.43	0.14	0.38	0.46	0.80			
(7) PE	0.90	0.56	0.45	0.14	0.27	0.22	0.46	0.88		
(8) SI	0.78	0.49	0.28	0.11	0.39	0.19	0.46	0.39	0.78	
(9) UB	0.78	0.52	0.43	0.21	0.49	0.43	0.60	0.41	0.41	0.66

N/A, not applicable (single-item construct).

All factor loadings were significant. The factor reliability values were above the recommended thresholds. Internal consistency was assessed via Cronbach's alpha coefficient, and all values were above 0.7, indicating respectable reliability for all constructs. Furthermore, the Fornell–Larcker ratio indicated the discriminant validity of the measurement.

Measurement quality was verified by examining convergent validity, discriminant validity and internal consistency.

Convergent validity was assessed as follows: Item reliability was inspected for each convergent item, and validity required indicator loadings to be 0.5 or more. The indicators had loadings above 0.5. Two items in the UB construct did not reach the threshold. However, to reflect the widely differing media types used in teaching, we decided to include media types with a lower impact on the latent UB construct. The remaining item loadings (see Table 3) demonstrated acceptable convergent validity and were retained for subsequent analysis.

For reflective models, outer loadings are the key indicators showing the trajectory of the latent variable towards the observed variables. Therefore, they show how much each observable variable or item contributes absolutely to the definition of the construct or latent variable.

Regarding discriminant validity, we compared all the items loaded for which we expected a higher value with the same construct compared to other variables (see Table 4). This comparison satisfied the discriminant validity criterion suggested by Chin (49).

**Table 4:** Cross-factor loadings and reliability of constructs.

Item	BI	EE	FC	FLO	ILO	PE	SI	UB
BI_1	**0.94**	0.42	0.41	0.30	0.56	0.55	0.49	0.53
BI_2	**0.73**	0.25	0.25	0.24	0.34	0.41	0.36	0.28
BI_3	**0.93**	0.41	0.40	0.32	0.56	0.51	0.42	0.52
EE_1	0.29	**0.86**	0.47	0.11	0.33	0.34	0.25	0.32
EE_2	0.39	**0.90**	0.50	0.13	0.38	0.44	0.27	0.38
EE_3	0.40	**0.86**	0.58	0.14	0.39	0.39	0.23	0.40
EE_4	0.39	**0.91**	0.53	0.09	0.41	0.42	0.26	0.40
FC_1	0.30	0.35	**0.87**	0.24	0.24	0.18	0.39	0.40
FC_2	0.42	0.78	**0.68**	0.18	0.45	0.39	0.28	0.39
FC_5	0.28	0.32	**0.87**	0.24	0.23	0.10	0.26	0.40
FLO	0.33	0.13	0.27	**1.000**	0.46	0.22	0.19	0.43
ILO_2	0.54	0.35	0.28	0.34	**0.83**	0.40	0.50	0.54
ILO_3	0.35	0.34	0.28	0.41	**0.73**	0.33	0.24	0.36
ILO_4	0.45	0.34	0.39	0.35	**0.82**	0.36	0.33	0.52
PE_1	0.51	0.42	0.31	0.22	0.41	**0.84**	0.36	0.39
PE_2	0.51	0.45	0.23	0.19	0.43	**0.91**	0.35	0.39
PE_3	0.45	0.37	0.22	0.23	0.38	**0.90**	0.31	0.32
PE_4	0.47	0.35	0.18	0.15	0.40	**0.86**	0.34	0.34
SI_1	0.38	0.22	0.21	0.18	0.44	0.38	**0.82**	0.30
SI_2	0.44	0.27	0.27	0.21	0.50	0.40	**0.83**	0.35
SI_3	0.35	0.18	0.36	0.05	0.23	0.18	**0.75**	0.31
SI_4	0.33	0.20	0.38	0.11	0.20	0.21	**0.71**	0.30
PC_1	0.42	0.22	0.35	0.35	0.47	0.39	0.34	**0.76**
PC_2	0.41	0.39	0.36	0.25	0.46	0.35	0.37	**0.74**
PC_3	0.30	0.24	0.29	0.21	0.33	0.18	0.22	**0.68**
PC_4	0.47	0.48	0.42	0.31	0.49	0.34	0.29	**0.78**
PC_5	0.34	0.34	0.37	0.41	0.46	0.24	0.33	**0.64**
PC_6	0.14	0.03	0.17	0.24	0.17	0.12	0.01	**0.47**
PC_7	0.04	0.07	0.09	0.24	0.14	0.03	−0.01	**0.43**
PC_8	0.29	0.86	0.47	0.11	0.33	0.34	0.25	**0.32**
PC_9	0.39	0.90	0.50	0.13	0.38	0.44	0.27	**0.38**

Bold values in a column belong to the construct in the column heading.

## 4 Results

**Figure 2:**
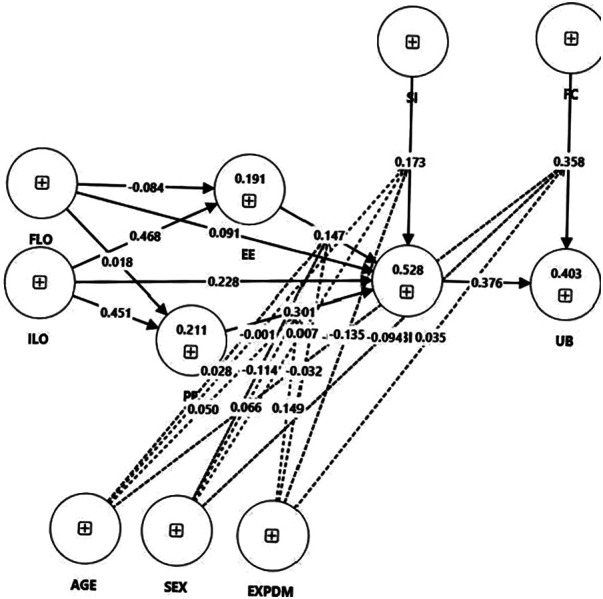
Effect of latent constructs: path diagram.

Bootstrapping was performed to provide the significance level for each hypothesised relationship. The parameter settings for bootstrapping included no sign changes and 5,000 samples. All results are summarised in Table 5. As illustrated in Figure 2, the impact of experience, FC and BI on UB was found to be significantly positive. Moreover, PE and SI showed significant effects on BI, as well as ILO on EE and PE. Hence, Hypotheses 1.2, 1.4, 1.6, 2.1.1, 2.2.1 and 2.3.1, as well as 3.1 and 3.1, were confirmed by the findings (Table 5). Regarding the moderating effects, it was determined that experience and age did not exhibit significant (*p* > .05) interactions with any of the constructs when considering all possible higher-order interactions. According to the data, age and gender showed no significant interaction at all; only experience showed a negative interaction with the direct effect of SI on BI. Therefore, only Hypothesis 2.3.4 could be confirmed.

**Table 5:** Summary of hypothesis testing.

Hypothesis		Path	Moderator	Effect	t-value	p-value	Result
1.1	Age has a negative effect on UB	Age → UB		−0.088	1.343	0.179	r Reject
1.2	There are no differences between genders in UB	Gender → UB		−0.169	1.572	0.116	Confirm
1.3	Experience has no effect on UB	Exp → UB		0.168	2.347	0.019	Reject
1.4	FC have a significant influence on UB	FC → UB		0.358	3.877	0.000	Confirm
1.5.1	The effect of FC on UB is stronger for older and more experienced teachers	FC → UB	Age	−0.113	1.908	0.056	Reject
1.5.2	The effect of FC on UB is stronger for more experienced teachers	FC → UB	Experience	0.035	0.440	0.660	Reject
1.6	BI has a significant influence on UB	BI → UB		0.376	6.680	0.000	Confirm
2.1.1	PE has a significant influence on BI	PE → BI		0.301	3.814	0.000	Confirm
2.1.2	The effect of PE on BI is larger for men than for women	PE → BI	Gender	0.066	0.541	0.589	Reject
2.1.3	The effect of PE on BI is larger for younger than for older teachers	PE → BI	Age	0.050	0.950	0.342	Reject
2.2.1	EE has a significant influence on BI	EE → BI		0.147	1.772	0.076	Confirm
2.2.2	The effect of EE on BI is larger for women than for men	EE → BI	Gender	−0.114	0.961	0.336	Reject
2.2.3	The effect of EE on BI is larger for older than for younger teachers	EE → BI	Age	0.028	0.423	0.672	Reject
2.2.4	The effect of EE on BI is larger for less experienced than for more experienced teachers	EE → BI	Experience	−0.032	0.448	0.654	Reject
2.3.1	SI has a significant influence on BI.	SI → BI		0.173	2.154	0.031	Confirm
2.3.2	The effect of SI on BI is larger for women than for men	SI → BI	Gender	0.007	0.054	0.957	Reject
2.3.3	The effect of SI on BI is larger for older than for younger teachers.	SI → BI	Age	−0.001	0.014	0.988	Reject
2.3.4	The effect of SI on BI is larger for less experienced than for more experienced teachers	SI → BI	Experience	−0.135	1.961	0.050	Confirm
3.1	ILO have a stronger positive effect on PE than FLO	ILO → PE		0.451	7.879	0.000	Confirm
		FLO → PE		0.018	0.349	0.727	
3.2	ILO have a stronger positive effect on EE than FLO	ILO → EE		0.468	8.947	0.000	Confirm
		FLO → EE		−0.084	2.027	0.043	

Standardised root mean square residual (SRMR) = 0.094; chi-square = 1,761.97.

## 5 Model fit

The determination coefficient R^2^ was between 0 and 1. The *R*^2^ value indicated the proportion of variance of the endogenous construct explained by all predecessor constructs associated with the endogenous construct. Moreover, 52.8% of the variance of BI was explained by the former construct (*R*^2^ = 0.528). This implied that 47.2% of the variance was not explained. For UB, EE and PE, *R*^2^ was 0.403, 0.191 and 0.211, respectively.

Before testing the structural model, fit adjustment with an SRMR value was evaluated. The result was an SRMR of 0.094, which indicated an acceptable fit adjustment.

## 6 Discussion

The findings of this study align with prior research on technology acceptance among educators, confirming the role of performance expectancy, social influence, facilitating conditions, and informal learning in shaping PE teachers' behavioural intention. However, beyond these individual and structural factors, revisiting the broader pedagogical justification for digital technology in PE is crucial. As discussed in the introduction, movement culture is increasingly shaped by digital tools, from tracking technologies to online movement tutorials. If PE aims to enable students to critically engage with contemporary movement practices (9), then teachers’ acceptance of these technologies is an essential prerequisite. Technology acceptance is not merely about ease of use or external expectations; instead, it is about ensuring that PE remains relevant to the evolving movement culture. This broader cultural perspective provides additional depth to the significance of the findings, particularly the strong influence of informal learning opportunities, which may reflect teachers' engagement with digital movement practices beyond formal training.

Concerning RQ1 (what influences the use behaviour of PE teachers with regard to digital technologies?), our data showed that the internal factor (total effect of BI on UB: 0.376) and the external factor (total effect of FC on UB: 0.358) influenced UB to almost the same extent. Nevertheless, approximately 60% of the variance of UB remained unexplained (*R*^2^ for UB = 0.403), which is comparable to studies using similar approaches (18). The hypothesised differences in technology use between different age levels (28) could not be supported by the data. This finding might have been due to the rapid spread of digital technologies. While in general research on technology acceptance, gender is assumed to influence technology acceptance (19), the data supported the finding from research on the technology use of teachers that there is no significant difference between genders (28). Moreover, contradicting the ICILS 2018 findings, there were differences between more and less experienced teachers concerning the use of digital technologies. In the sample of this study, PE teachers who were more experienced in using digital technologies used digital technologies significantly more often than less experienced teachers. The data could not confirm the moderating effects of age and experience as hypothesised by the UTAUT model (19).

Concerning RQ2 (What influences the behavioural intention of PE teachers towards digital technologies?), the data showed that the variance of BI was explained to 53.8%, with significant influences from PE and SI. Unexpected EE did not show a significant influence. This non-significant result may indicate that ease of use is no longer a decisive factor for this group of teachers—possibly due to a generally high baseline of digital competence—highlighting the need for future research that includes differentiated measures of digital literacy to explain variance in Effort Expectancy effects. The hypothesised moderation effects of age, gender and experience on the direct effects of PE, EE and SI on BI could not be confirmed by the data, with the exception of the light negative effect of experience on the direct effect of SI on BI. It seemed that teachers who were less experienced in using digital technologies were more dependent on their intention to use digital technologies than their more experienced colleagues. The non-significant effect of EE suggested that teachers in the sample might already have possessed a high baseline level of digital literacy, making perceived ease of use a less relevant determinant of their technology adoption. A further possible explanation for the lack of a significant EE effect is that PE teachers may prioritise pedagogical value over usability, as indicated by data on motives for professional development (50). While ease of use can be a barrier in some contexts, PE teachers may be more motivated by whether digital tools effectively support skill acquisition, student engagement and movement-related learning outcomes. This interpretation is supported by the strong effect of performance expectancy, indicating that teachers' decisions to adopt technology are primarily driven by perceived educational benefits rather than ease of use.

The unexpected missing effects of the moderating variables (especially age and gender) might be explained by recent data indicating that technology use is now more institutionalised and widely accepted (51, 52), contrasting with the findings of earlier studies [e.g., (19)].

Concerning RQ3 (Do different learning opportunities influence the internal factors relating to the technology acceptance of physical education teachers?), the data clearly showed that informal learning opportunities, such as interaction with colleagues, reading literature, and reflecting on one's practice, had a stronger impact on the performance and effort expectancy towards digital tools than formal learning opportunities. While we found highly significant and strong effects of informal learning opportunities on performance expectancy and effort expectancy, formal learning opportunities showed no effect on performance expectancy and a light negative effect on effort expectancy. The advantage of ILO is in line with what was hypothesised, but surprisingly, FLO showed no effect or even a light negative effect (on EE). This apparent difference might be explained by approaches that consider the situatedness of teacher knowledge acquisition (37, 53). According to this perspective, it is an enormous challenge for teachers to transfer rather abstract knowledge as it is often presented in formal development settings to concrete contexts, such as planning for a concrete learning group. Informally acquired knowledge is *per se* bound to a concrete problem relating to work–life; therefore, no transfer is needed (33, 34). The missing impact of FLO might have been due to the low quality of the FLO measures. This aspect might be addressed by FLO components that regard the quality of the learning opportunities and the engagement of the teachers who participate.

## 7 Conclusion, limitations and future research

If it is reasonable to support teachers’ use of digital technologies, two results of this study might guide interventions. First, personal internal and external factors are equally relevant. In other words, a willing teacher needs to have an appropriate environment, and a well-equipped environment needs to have willing teachers. Therefore, discussions about priorities in fostering digitalisation in schools should not focus on the teachers or the hardware but equally on both aspects.

Second, it seems worthwhile to support informal learning opportunities for teachers to influence their behavioural intentions towards using digital technologies. The hesitant debates about recognising and promoting informal learning may provide an indication that fostering informal learning is not easy to implement. Although much more research is needed to understand the complex nature of informal learning, two key factors for fostering informal learning are time to reflect on one's own practice individually and in communities of practice and high-quality materials and products that support working processes such as lesson planning. To strengthen teacher development, future initiatives should incorporate structured support for informal learning, such as peer exchange formats and integrated time for collaborative reflection.

Several methodological limitations should be considered. First, the sample that was reached (*N* = 239 teachers) did not represent all PE teachers in the research area. According to the Statistical Bureau, of North Rhine–Westfalia, the federal state has a total of 180,340 teachers (54). Moreover, based on the graduation numbers in university teacher education (the proportion of graduates in PE is 3.9%; (55), the population size is 7,033. With a confidence interval of 95% and a margin error of 5%, a minimum of 365 teachers would build a representative sample. Second, recruiting via social media is known to be selective and, therefore, biased. An age bias, as discussed by Darko, Klieb, and Olson (56), was not indicated by the sample's descriptive data, with participants having a mean age of 41.9. Furthermore, it might be argued that the sample contained more people using digital technologies intensely due to social media recruitment. Such a bias was not indicated by the descriptive data. The ICILS showed a 69.9% rate of teachers using digital technologies daily (over all subjects; (51). In our sample, the teachers only indicated a 40.2% rate for their weekly usage. The lower rate might be seen as an indicator of not having a pro-digital technology usage bias in the dataset.

A third methodological limitation was the use of self-reported data for the UB construct, as these kinds of data are known to be susceptible to social desirability bias. This problem has also been reported in relation to working time analysis for teachers (57). This might have biased the dataset in terms of higher UB values. For economic reasons, other methods, such as observations or time–use protocols, were not an option.

An important future research perspective is to deepen the understanding of teachers' technology acceptance and its potential to influence learning processes in the workplace. Given the assumption that digital technologies, such as databases, feedback tools, and AI agents, have the potential to be learning opportunities for teachers, it is essential to gain a better understanding of the relationship between utilisation/learning processes and learning outcomes. It must be assumed that different technologies bear different learning potentials and have different impacts on professional competences. In future studies, other components of professional competence, such as self-efficacy (58), teacher beliefs (59), and digital competences (60), might be considered to map learning outcomes. Moreover, future studies could examine the role of institutional support, especially the role of the school administration (50), as an additional factor influencing BI and UB.

Furthermore, the unexplained variance of BI and UB indicates additional missing factors relating to technology acceptance. In future research, other constructs, such as attitude towards technology (58) and perceived risk (61), might be considered to reach higher levels of explanation.

## Data Availability

The raw data supporting the conclusions of this article will be made available by the authors, without undue reservation.

## References

[1] Casey A Goodyear VA Armour K, editors. Digital Technologies and Learning in Physical Education: Pedagogical Cases. London: Routledge (2017).

[2] Jastrow F Greve S Thumel M Diekhoff H Süßenbach J. Digital technology in physical education: a systematic review of research from 2009 to 2020. Ger J Exerc Sport Res. (2022) 52:504–28. 10.1007/s12662-022-00848-5

[3] van Hilvoorde I Koekoek J. Next generation PE: thoughtful integration of digital technologies. In: van Hilvoorde I Koekoek J, editors. Digital Technology in Physical Education: Global Perspectives. London: Routledge (2018). p. 1–15.

[4] Stalder F. The Digital Condition. Cambridge: Polity (2018).

[5] Rode D. Digitalisierung als kultureller prozess: grundlegende bestimmungen und sportpädagogische anschlüsse jenseits der technologie [Digitalization as a cultural process: fundamental principles and sportpedagogical implications beyond technology]. In: Steinberg C Bonn B, editors. Digitalisierung und Sportwissenschaft. Baden-Baden: Academia (2021). p. 9–62.

[6] Wibowo J Genfeld L Hofmann R Wolters H. Digitale Tools und Digitalität im Sportunterricht als Bedingungen von Bewegungsbildung [Digital Tools and Digitality in Physical Education as Prerequisites for Movement Bildung.]. In: Balz E Bindel T, editors. Bildungszugänge im Sport. Wiesbaden: Springer (2023). 147–62. 10.1007/978-3-658-38895-9_12

[7] Brinkmann M Giese M. Practising the practice. Towards a theory of practising in physical education from a bildung-theoretical perspective. Phys Educ Sport Pedagogy. (2021) 30:50–63. 10.1080/17408989.2023.2167968

[8] Madsen KL Aggerholm K. Embodying education: a Bildung theoretical approach to movement integration. Nord J Stud Educ Policy. (2020) 6:157–64. 10.1080/20020317.2019.1710949

[9] Wibowo J Krieger C Gaum C Dyson B. Bildung: a German student-centered approach to health and physical education. Eur Phy Educ Rev. (2023) 29:233–50. 10.1177/1356336X221133060

[10] Ruin S Stibbe G. Health-oriented “Bildung” or an obligation to a healthy lifestyle? A critical analysis of current PE curricula in Germany. Curriculum J. (2021) 32:136–51. 10.1002/curj.92

[11] Anderson LW. Instruction and time-on-task: a review. J Curriculum Stud. (1981) 13:289–303. 10.1080/0022027810130402

[12] Herrmann C Gerlach E. Unterrichtsqualität im fach sport—ein Überblicksbeitrag zum forschungsstand in theorie und empirie [Instructional quality in physical education—an overview of the current state of research in theory and practice]. Unterrichtswissenschaft. (2020) 48:361–84. 10.1007/s42010-020-00080-w

[13] Wibowo J Krieger C Gerlach E Bükers F. Wie generisch, fachspezifisch oder inhaltsspezifisch ist aktivierung im fach sport? [How generic, subject-specific, or content-specific is activation in Physical Education?]. In: Wibowo J Krieger C Gerlach E Bükers F, editors. Aktivierung im Sportunterricht. Hamburg: Universität Hamburg (2024). p. 263–92.

[14] Munzert J Lorey B Zentgraf K. Cognitive motor processes: the role of motor imagery in the study of motor representations. Brain Res Rev. (2009) 60:306–26. 10.1016/j.brainresrev.2008.12.024 19167426

[15] Wibowo J Dyson B. A contingency perspective on learning and instruction in physical education. Eur Phy Educ Rev. (2021) 27:727–42. 10.1177/1356336X20985884

[16] Voogt J Pareja Roblin N. A comparative analysis of international frameworks for 21st century competences: implications for national curriculum policies. J Curriculum Stud. (2012) 44:299–321. 10.1080/00220272.2012.668938

[17] Tan M Subramonyam H. More Than Model Documentation: Uncovering Teachers’ Bespoke Information Needs for Informed Classroom Integration of ChatGPT. Ithaca, NY: Cornell University. (2023). Available online at: https://arxiv.org/abs/2309.14458

[18] Scherer R Siddiq F Tondeur J. The technology acceptance model (TAM): a meta-analytic structural equation modeling approach to explaining teachers’ adoption of digital technology in education. Comput Educ. (2019) 128:13–35. 10.1016/j.compedu.2018.09.009

[19] Venkatesh V Morris MG Davis GB. User acceptance of information technology: toward a unified view. MIS Q. (2003) 27:425–78. 10.2307/30036540

[20] Kułak JP Trojanowski M Barmentloo E. A literature review of the partial unified theory of acceptance and use of technology 2 (UTAUT2) model. Ann Univ Mariae Curie-Skłodowska Sect H—Oeconomia. (2019) 53:101–13. 10.17951/h.2019.53.4.101-113

[21] Frank M Milković V. Determinants of technology acceptance: an empirical study based on the unified theory of acceptance and use of technology. J Serv Manag. (2018) 29:555–75. 10.1108/JOSM-03-2018-0074

[22] Granić A Marangunić N. Technology acceptance model in educational context: a systematic literature review. Br J Educ Technol. (2019) 50:2572–93. 10.1111/bjet.12864

[23] Killian CM Goad T Daum DN Gentry C. Exploring K-12 physical education teachers’ acceptance and use of online learning: a theoretical approach. Am J Dist Educ. (2025):1–19. 10.1080/08923647.2025.2482290

[24] Robinson DB Randall L. Marking physical literacy or missing the mark on physical literacy? A conceptual critique of Canada’s physical literacy assessment instruments. Meas Phys Educ Exerc Sci. (2017) 21:40–55. 10.1080/1091367X.2016.1249793

[25] Wyant, J Baek Y. Teachers’ perceptions of digital technology integration: assessing feasibility, competence, and support. Educ Technol Res Dev. (2018) 66(5):1237–55. 10.1007/s11423-018-9576-3

[26] Drossel K Gerick J Niemann J Eickelmann B Domke M. Die perspektive der lehrkräfte auf das lehren mit digitalen medien und die förderung des erwerbs computer- und informationsbezogener kompetenzen in deutschland im internationalen vergleich [Teachers’ perspectives on teaching with digital media and promoting the acquisition of computer and information-related skills in Germany in an international comparison]. In: Eickelmann B Fröhlich N Bos W Gerick J Goldhammer F Schaumburg H, editors. ICILS 2023 #Deutschland Computer- und Informationsbezogene Kompetenzen und Kompetenzen im Bereich Computational Thinking von Schüler*innen im Internationalen Vergleich. Münster: Waxmann (2024). p. 149–87.

[27] Vennemann M Eickelmann B Labusch A Drossel K. ICILS 2018 #Deutschland. Dokumentation der Erhebungsinstrumente der Zweiten Computer and Information Literacy Study [ICILS 2018 #Germany. Documentation of the Survey Instruments Used in the Second Computer and Information Literacy Study.]. Münster: Waxmann (2021).

[28] Drossel K Eickelmann B Schaumburg H Labusch A. Nutzung digitaler medien und prädiktoren aus der perspektive der lehrerinnen und lehrer im internationalen vergleich [Use of digital media and predictors from the perspective of teachers in an international comparison]. In: Eickelmann B Bos W Gerick J Goldhammer FH Schaumburg K Schwippert M, editors. ICILS 2018 #Deutschland. Computer- und Informationsbezogene Kompetenzen von Schülerinnen und Schülern im Zweiten Internationalen Vergleich und Kompetenzen im Bereich Computational Thinking. Münster: Waxmann (2019). p. 205–40.

[29] Inan FA Lowther DL. Factors affecting technology integration in K–12 classrooms: a path 2del. Educ Technol Res Dev. (2010) 58:137–54. 10.1007/s11423-009-9132-y

[30] Lumpe AT Chambers E. Assessing teachers’ context beliefs about technology use. J Res Technol Educ. (2001) 34:93–107. 10.1080/15391523.2001.10782337

[31] Richter D. Professional development across the teaching career. In: Kunter M Baumert J Blum W Klusmann U Krauss S Neubrand M, editors. Cognitive Activation in the Mathematics Classroom and Professional Competence of Teachers. Boston: Springer. (2013). 333–42. 10.1007/978-1-4614-5149-5_17

[32] Dron J Anderson T. Informal learning in digital contexts. In: Zawacki-Richter O Jung I, editors. Handbook of Open, Distance and Digital Education. Singapore: Springer (2023). 1373–89. 10.1007/978-981-19-2080-6_84

[33] Kyndt E Gijbels D Grosemans I Donche V. Teachers’ everyday professional development. Rev Educ Res. (2016) 86:1111–50. 10.3102/0034654315627864

[34] Wibowo J. Informal learning processes in the daily practice of one German PE teacher. Phys Educ Sport Pedagogy. (2023) 1–13. 10.1080/17408989.2023.2260396

[35] Livingston DW. Informal learning: conceptual distinctions and preliminary findings. Counterpoints. (2006) 249:203–27.

[36] Stern E Sommerlad E. Workplace Learning, Culture and Performance. Institute of Personnel and Development (1999).

[37] Armour KM Yelling M. Effective professional development for physical education teachers: the role of informal, collaborative learning. J Teach Phys Educ. (2007) 26:177–200. 10.1123/jtpe.26.2.177

[38] Neumann P Zimmermann R. Zur akzeptanz von sitzunterbrechungen im unterricht aus der perspektive von lehrkräften [Teachers’ acceptance of interruptions during lessons]. Sportunterricht. (2020) 69:2–9.

[39] Patton K Griffin LL. Three physical education programs’ adaptive approaches to change: “how can I spin that so it works for me?”. Sport Educ Soc. (2008) 13:413–30. 10.1080/13573320802445033

[40] Dohmen G. Das Informelle Lernen [Informal Learning]. Bonn: BMBF Publik (2001).

[41] Marsick VJ Watkins K. Informal and Incidental Learning in the Workplace. London: Routledge (1990).

[42] Nistor N Wagner M Heymann JO. Prädiktoren und moderatoren der akzeptanz von bildungstechnologien. Die unified theory of acceptance and use of technology auf dem prüfstand [Predictors and moderators of the acceptance of educational technologies. The unified theory of acceptance and use of technology put to the test.]. Empirische Pädagogik. (2012) 26:343–71. 10.4324/9781315715926

[43] Urban D Mayerl J. Strukturgleichungsmodellierung [Structural equation modeling]. Wiesbaden: Springer. (2014). 10.1007/978-3-658-01919-8

[44] Weiber R Sarstedt M. Strukturgleichungsmodellierung [Structural equation modeling]. Wiesbaden: Springer. (2021). 10.1007/978-3-658-32660-9

[45] Ringle CM Wende S Becker JM. SmartPLS 3. Bönningstedt: SmartPLS GmbH. (2015). Available online at: https://www.smartpls.com

[46] Chin WW. The partial least squares approach for structural equation modeling. In: Marcoulides GA, editor. Modern Methods for Business Research. New York: Lawrence Erlbaum Associates (1998). p. 295–336.

[47] Hair JF Matthews LM Matthews RL Sarstedt M. PLS-SEM or CB-SEM: updated guidelines on which method to use. Int J Multivar Data Anal. (2017) 1:107–23. 10.1504/IJMDA.2017.087624

[48] Blanz M. *Forschungsmethoden und Statistik für die Soziale* [Research methods and statistics for social sciences]. Kohlhammer. (2015). 10.17433/978-3-17-025836-5

[49] Chin WW. How to write up and report PLS analyses. In: Esposito Vinzi V Chin WW Henseler J Wang H, editors. Handbook of Partial Least Squares: Concepts, Methods and Applications in Marketing and Related Fields. Stuttgart: Springer (2010). p. 655–90.

[50] Richter D Kleinknecht M Gröschner A. What motivates teachers to participate in professional development? An Empirical Investigation of Motivational Orientations and the Uptake of Formal Learning Opportunities. Teach Teacher Educ. (2019) 86:102929. 10.1016/j.tate.2019.102929

[51] Eickelmann B Fröhlich N Bos W Gerick J Goldhammer F Schaumburg H, editors. ICILS 2023 #Deutschland. Computer- und Informationsbezogene Kompetenzen und Kompetenzen im Bereich Computational Thinking von Schüler*innen im Internationalen Vergleich [ICILS 2023 #Germany. Computer and Information-related skills and Computational Thinking Skills of Students in International Comparison]. Münster: Waxmann. (2024). 10.31244/9783830999492

[52] Kipp M. Impact of the COVID-19 pandemic on the acceptance and use of an E-learning platform. Int J Environ Res Public Health. (2021) 18:11372. 10.3390/ijerph182111372 34769887 PMC8582837

[53] Kwakman K. Factors affecting teachers’ participation in professional learning activities. Teach Teacher Educ. (2003) 19:149–70. 10.1016/S0742-051X(02)00101-4

[54] Statistisches Landesamt NRW. (2025, o. J.). Lehrkräfte in NRW 2024/2025 [Teachers in North Rhine-Westphalia 2024/2025]. Available online at: https://statistik.nrw/schulen-nordrhein-westfalen#pts_4803

[55] Statistisches Bundesamt. (2023). Bildung und Kultur. Studierende an Hochschulen: Fachserie 11 Reihe 4.1. Sommersemester 2022 [Education and culture. Students at universities: Specialist series 11, series 4.1. Summer semester 2022]. Available online at: https://www.statistischebibliothek.de/mir/servlets/MCRFileNodeServlet/DEHeft_derivate_00076045/2110410227314.pdf

[56] Darko EM Kleib M Olson J. Social Media use for research participant recruitment: integrative literature review. J Med Internet Res. (2022) 24:e38015. 10.2196/38015 35925655 PMC9389385

[57] te Braak P van Droogenbroeck F Minnen J van Tienoven TP Glorieux I. Teachers’ working time from time–use data: consequences of the invalidity of survey questions for teachers, researchers and policy. Teach Teacher Educ. (2022) 109:103536. 10.1016/j.tate.2021.103536

[58] Pan X. Technology acceptance, technological self-efficacy, and attitude toward technology-based self-directed learning: learning motivation as a mediator. Front Psychol. (2020) 11:564294. 10.3389/fpsyg.2020.564294 33192838 PMC7653185

[59] Ertmer PA Ottenbreit-Leftwich A Tondeur J. Teacher beliefs and uses of technology to support 21st century teaching and learning. In: Fives H Gill MG, editors. International Handbook of Research on Teachers’ Beliefs. London: Routledge (2015). p. 403–18.

[60] Skantz-Åberg E Lantz-Andersson A Lundin M Williams P. Teachers’ professional digital competence: an overview of conceptualisations in the literature. Cogent Educ. (2022) 9:2063224. 10.1080/2331186X.2022.2063224

[61] Chao CM. Factors determining the behavioral intention to use mobile learning: an application and extension of the UTAUT model. Front Psychol. (2019) 10:1652. 10.3389/fpsyg.2019.01652 31379679 PMC6646805

