# Supplementary material for: Predicting the power of informal learning opportunities on technology use of physical education teachers
Source: Front Sports Act Living. 2025 Oct 20;7:1653809. doi: 10.3389/fspor.2025.1653809 (PMC12580269; doi:10.3389/fspor.2025.1653809)
Supplement: Supplementary file 1 [file Table1.docx]

Appendix 1 to

Wibowo, J., Hofmann, R., Brand, T. & Wiese, H. (2025). Predicting the power of informal learning opportunities on technology use of physical education teachers. *Frontiers in sports and active living*, *7.* https://doi.org/10.3389/fspor.2025.1653809

| Item# | Itemtext (german, used in the questionnaire) | Itemtext (English) |
| --- | --- | --- |
| FLO | Bitte geben Sie an, an wie vielen Fortbildungen Sie in den vergangen zwei Jahren zum Thema digitale Tools im Sportunterricht teilgenommen haben? | Please indicate how many training sessions on the topic of digital tools in PE lessons you have attended in the past two years |
| ILO1 | Wie oft sprechen Sie mit Kolleg:innen über den Einsatz digitaler Tools im Sportunterricht? | How often do you talk with colleagues about the use of digital tools in PE lessons? |
| ILO2 | Wie oft greifen Sie auf Fachliteratur oder andere Quellen zurück, um etwas über digitale Tools im Sportunterricht zu lernen? | How often do you consult professional literature or other sources to learn about digital tools in PE lessons? |
| ILO3 | Wie oft reflektieren Sie den Einsatz von digitalen Tools im Sportunterricht nach dem Unterricht? | How often do you reflect on the use of digital tools in PE lessons after teaching? |
| UB1-9 | Geben Sie dazu bitte an, wie häufig Sie die folgenden digitalen Tools in Ihrem Sportunterricht einsetzen:   1. Präsentationstools (z. B. Powerpoint, Prezi) 2. Lernmanagementsysteme (z. B. Moodle, ItsLearning) 3. Cloudspeicher (z. B. Dropbox, OneDrive) 4. Produktionstools (z. B. Kamera-Apps, Office-Apps) 5. Bewegungsbezogene Tools (z. B. Coaches Eye, Freeletics) 6. Recherchetools (z. B. Wikipedia, Google-Suche) 7. Social Media (z. B. TikTok, Instagram) 8. Messenger (z. B. WhatsApp, Signal) 9. Orientierungstools (z. B. GoogleMaps, Komoot) | Please indicate how often you use the following digital tools in your physical education classes:   1. Presentation tools (e.g., PowerPoint, Prezi) 2. Learning management systems (e.g., Moodle, ItsLearning) 3. Cloud storage (e.g., Dropbox, OneDrive) 4. Production tools (e.g., camera apps, Office apps) 5. Movement-related tools (e.g., Coaches Eye, Freeletics) 6. Research tools (e.g., Wikipedia, Google Search) 7. Social media (e.g., TikTok, Instagram) 8. Messengers (e.g., WhatsApp, Signal) 9. Orientation tools (e.g., Google Maps, Komoot) |
| BI1 | Ich beabsichtige digitale Tools in den nächsten Monaten zu nutzen. | I intend to use digital tools in the coming months. |
| BI2 | Ich rechne damit, digitale Tools in den nächsten Monaten zu nutzen. (Habe jedoch keinen direkten Einfluss darauf) | I expect to use digital tools in the coming months (but I do not have direct control over this). |
| BI3 | Ich habe konkrete Pläne, digitale Tools in den nächsten Monaten zu nutzen. | I have concrete plans to use digital tools in the coming months. |
| PE1 | Digitale Tools sind für meinen alltäglichen Unterricht nützlich. | Digital tools are useful for my everyday teaching. |
| PE2 | Mit Hilfe von digitalen Tools kann das Lernen der Schüler:innen besser gefördert werden. | With the help of digital tools, student learning can be better supported. |
| PE3 | Mit digitalen Tools kann ich die Produktivität im Unterricht erhöhen. | With digital tools, I can increase productivity in lessons. |
| EE4 | Durch die Nutzung digitaler Tools kann ich die Motivation meiner Schüler:innen fördern. | By using digital tools, I can foster my students’ motivation. |
| EE1 | Die Bedienung von digitalen Tools für unterrichtliche Zwecke ist im Allgemeinen eindeutig und verständlich. | Operating digital tools for teaching purposes is generally clear and understandable. |
| EE2 | Im Allgemeinen kann ich schnell lernen, digitale Tools im Unterricht geschickt einzusetzen. | In general, I can quickly learn to use digital tools effectively in lessons. |
| EE3 | Die Verwendung von digitalen Tools im Sportunterricht finde ich generell eher leicht. | I generally find it rather easy to use digital tools in PE lessons. |
| EE4 | Im Allgemeinen fällt es mir leicht, die Bedienung von digitalen Tools für den Sportunterricht zu erlernen. | In general, I find it easy to learn how to operate digital tools for PE lessons. |
| SI1 | Personen, die Einfluss auf mein Verhalten haben, sind der Meinung, ich sollte digitale Tools benutzen. | People who influence my behavior think I should use digital tools. |
| SI2 | Personen, die mir wichtig sind, sind der Meinung, ich sollte digitale Tools benutzen. | People who are important to me think I should use digital tools. |
| SI3 | Die Schulleitung unterstützt die Nutzung von digitalen Tools. | The school leadership supports the use of digital tools. |
| SI4 | Im Allgemeinen unterstützt meine Schule die Nutzung von digitalen Tools. | In general, my school supports the use of digital tools. |
| FC1 | Ich habe alle organisatorischen Voraussetzungen, um digitale Tools zu nutzen. | I have all the organizational conditions necessary to use digital tools. |
| FC2 | Ich habe das Wissen, das notwendig ist, um digitale Tools zu nutzen. | I have the knowledge necessary to use digital tools. |
| FC3 | Die Verwendung digitaler Tools passt zu meinem Unterrichtsstil. | The use of digital tools fits my teaching style. |
| FC4 | Eine bestimmte Person/Gruppe ist ansprechbar, wenn ich Probleme dabei habe, digitale Tools zu benutzen. | A specific person/group is available when I have problems using digital tools. |
| FC5 | Ich habe alle technischen Voraussetzungen, um digitale Tools zu nutzen. | I have all the technical conditions necessary to use digital tools. |
